# The red/blue light ratios from light-emitting diodes affect growth and flower quality of *Hippeastrum hybridum* ‘Red Lion’

**DOI:** 10.3389/fpls.2022.1048770

**Published:** 2022-12-01

**Authors:** Shunli Wang, Xiaoting Liu, Xiaoning Liu, Jingqi Xue, Xiuxia Ren, Yanning Zhai, Xiuxin Zhang

**Affiliations:** Key Laboratory of Biology and Genetic Improvement of Flower Crops (North China), Ministry of Agriculture and Rural Affairs, Institute of Vegetables and Flowers, Chinese Academy of Agricultural Sciences, Beijing, China

**Keywords:** *Hippeastrum*, blue light, red light, LEDs, photosynthesis, chlorophyll florescence, flowering

## Abstract

Light quality strongly impacts the growth and flower quality of ornamental plants. The optimum light quality for the growth and flowering of *Hippeastrum* remains to be validated. In the present study, we investigated the effect of the red/blue light ratio of LEDs on the growth and flowering quality of *H. hybrid* ‘Red Lion’. Two LEDs with red/blue light ratio of 1:9 (R_10_B_90_) and 9:1 (R_90_B_10_) were designed. LEDs of white light were the control. In the earlier vegetative and reproductive growth phase, R_90_B_10_ increased the biomass of the bulbs, leaves, and flowers. Compared with the control and R_10_B_90_ group, R_90_B_10_ LEDs delayed flowering by 2.30 d and 3.26 d, respectively. Based on chlorophyll contents, photosynthetic capacity, chlorophyll fluorescence parameters, and carbohydrate contents, the photosynthesis rate was higher in the R_10_B_90_ group. Optimal red and blue light intensity promoted the accumulation of carbohydrates and early flowering and prolonged the flowering period of *H. hybrid*. Microscopic analysis showed that stomatal density was high, and the number of chloroplasts was large in the R_10_B_90_ treatment group, which enhanced photosynthesis. Particularly, R_10_B_90_ promoted the expression of seven key genes related to chlorophyll synthesis. R_10_B_90_ also promoted early overexpression of the *HpCOL* gene that promotes early flowering. Thus, higher blue light and 10% red light intensities promote early and extended flowering, while higher red light and 10% blue light promote vegetative plant growth but delay flowering.

## Introduction

The *Hippeastrum* genus, also referred to as *Amaryllis*, originated from the tropical and subtropical regions of Central and South America, mainly in Brazil, Peru, and Bolivia. Members of this genus are monocotyledonous plants in the Amaryllidaceae family, comprising 75 species (Wang et al., 2018). Most members of the *Hippeastrum* genus have large and colorful flowers of ornamental value (Byamukama et al., 2006). Amaryllis is a favourite flower for Christmas and New Year decorations worldwide (Silberbush et al., 2003). *Hippeastrum* hybrids were introduced into China at the beginning of the 20th century, and after cultivar screening for better ornamental characteristics, domestication, and cultivation over time, they have been adapted in many provinces in China, especially in Yunnan and Guangdong (Shi et al., 2014). Many cut and potted amaryllis flowers are popular in China and are always part of the serving during the Chinese Spring Festival. However, the low temperature in North China in the winter season does not favor the cultivation of Amaryllis in the region. Likewise, forcing culture of Amaryllis is always used to achieve year-round flowering because its flowering time can easily be manipulated (De Hertogh and Gallitano, 2000).

Genetic and environmental factors influence the growth, development, and reproduction of higher plants (Fantini and Facella, 2020). Light quantity and quality are important environmental factors that influence numerous plant processes, including photosynthesis, germination, flowering, and several other metabolic/physiological processes light (Ramalho et al., 2002). Light quality, quantity, periodicity, and duration are reflected through the content of photosynthetic pigments and the expression of genes for various photoreceptors (Thomas, 2006; Fantini and Facella, 2020). Red light is absorbed by billin-containing phytochromes, whereas blue light is absorbed by flavin-containing cryptochromes and/or phototropin (Lin, 2000; Chaves et al., 2011; Petroutsos et al., 2016). Red (R) and blue (B) light influences plant shoot and stem elongation, and flowering in petunia, rose, and poinsettia (Islam et al., 2012; Abidi et al., 2013; Terfa et al., 2013; Gautam et al., 2015; Fukuda et al., 2016). Supplementary blue light increases the biomass and yield of tomatoes grown in the greenhouse (Kaiser et al., 2019). Manipulation of light quality with artificial light systems has been used to improve the production of commercial crops.

Light-emitting diodes (LEDs) have several advantages over natural light, including solid-state, long-lasting, and provide a narrow light spectrum suitable for flowering and metabolism of many horticultural plants, both of which increase the yield and improve the quality of the products (Gautam et al., 2015; Kaiser et al., 2019; Naznin et al., 2019; Virsile et al., 2020). Therefore, it is important to investigate how LED light affects plant growth and development. Previous studies showed that blue and red lights from LEDs enhance the growth, pigmentation, and antioxidant capacity of horticultural crops (Naznin et al., 2019). However, the best combination of red and blue light for the optimal growth and flowering of members in the *Hippestrum* genus is still unclear.

Four red/blue light ratios, including a red/blue light ratio of 9:1 (R_90_B_10_), 7:3 (R_70_B_30_), 5:5 (R_50_B_50_), 1:1:1 (R_33_B_33_W_33_), 1:9 (R_10_B_90_), and white light, affected growth and development of *H. hybridum* ‘Red Lion’ were investigated in our lab. It was showed that R_90_B_10_ and R_10_B_90_ significantly affect the growth and flowering quality of *H.* × *hybridum*. However, the precise mechanisms underlying this process are unclear.

In the present study, the effect of the red/blue light ratio of LEDs on the growth and flowering quality of Amaryllis under forcing-culture was investigated. Two LEDs with a red/blue light ratio of 9:1 (R_90_B_10_) and 1:9 (R_10_B_90_) were used. White LED light (at a color temperature of 6500 K) was used as the control. The effect of the red/blue LED light on the morphological characteristics, photosynthesis rate, chlorophyll content, physiological substance contents, and flowering quality of Amaryllis were investigated. The effect of the red/blue LED light on chlorophyll fluorescence as well as stomatal and chloroplast ultrastructure of leaves was also investigated using a microscope. The effect of the red/blue LED light on the expression of chlorophyll synthesis and flowering-related genes was analyzed using qRT-PCR. The findings of this research provide practical evidence on how to improve the cultivation of Amaryllis in a controlled environment.

## Materials and methods

### Plant materials and growth conditions

A total of 300 *H.* × *hybridum* ‘Red Lion’ bulbs were purchased from Beijing Dadongliu Nursery, Beijing, China. Of these, 270 bulbs with 17 - 19 cm circumference were selected for the experiments. The bulbs were stored at 4 ± 1°C for about 50 days to break dormancy. After pre-chilling, the bulbs were planted in plastic containers 20 cm wide in peat and perlite substrates mixed at the ratio of 2: 1. The 270 bulbs were randomly divided into three groups (n = 90 each) for further experiments. Before planting, the outer black-brown scales, wilted roots, and damaged tissues of the basal plate were removed. The bulbs were then soaked in 0.1% carbendazol solution for 40 min and wiped off using sterile tissue paper. The potted bulbs were incubated in a growth chamber under three different light quality conditions. The experiment was performed at the Institute of Vegetables and Flowers, Chinese Academy of Agricultural Sciences in Beijing, China. The day/night temperature was maintained at 23 ± 2°C, and the air humidity was about 65% - 80%.

### LED light treatments

Two light spectra of 90% red plus 10% blue LEDs (R_90_B_10_) and 10% red plus 90% blue LEDs (R_10_B_90_) were used in this study, selected according to the findings of previous studies. White LED (color temperature of 6500 K) was the control. Each treatment comprised three replications, each 30 pots. The photoperiod was 14 h, and photosynthetic photo flux density (PPFD) was 200 ± 5 µmol m^-2^ s^-1^. The LED lights were designed by EBIOSM Biotechnology CO. Ltd. (Beijing, China), and they consisted of 1.78 m × 0.08 m × 0.02 m linear fixtures, which were an array of five LEDs. The culture shelf height was 1.2 m. The photoperiod was 16 h of light and 8 h darkness. A light-shielding cloth was used in each treatment to prevent the effect of outside light.

### Measurements of morphological characteristics of ‘Red Lion’

After bulb planting, the morphological characteristics, including plant height, bulbs diameter, leaf number, leaf length, leaf width, the ratio of leaf length, and width, among others, of ‘Red Lion’ Amaryllis were recorded, and plant morphological changes were also photographed every 14 d for six phases, on 0 d, 14 d, 28 d, 42 d, 56 d and 72 d. Each measurement and the experiments were conducted in triplicate.

To determine the effects of red and blue light quality on the flowering quality of ‘Red Lion’, the flower number, length, width and diameter, scape number and diameter, initial flowering period (days at the first flower opening), flower period of a single flower, each scape (days from the first flower opening to the last flower withered of each scape) and each whole plant (days from the first flower opening to the last flower withered of each plant), plant height at the blooming stage, and the ratio of flower and leaf number were measured after 42 d. For every parameter, at least nine plants were randomly selected for each treatment to be measured.

### Measurements of petal chromatic aberration

The petal color chromatic aberration of ‘Red Lion’ at the blooming stage (42 d - 56 d) in each treatment was measured with a hand-held color analyzer (CM-2500D, Konica Minolta, Japan) in a room, according to the CIE L*a*b* system. The petals were collected from at least three plants, and the exterior, middle, and interior of the petals were measured separately, and the average value was used in the subsequent analyses.

### Measurement of chlorophyll content, photosynthetic capacity, and chlorophyll fluorescence parameters

The relative chlorophyll content in ‘Red Lion’ leaf from 14 d - 70 d was determined with SPAD-502 chlorophyll content analyzer (Shimadzu Corp., Kyoto, Japan). Three potted plants for each treatment were randomly selected, and three leaves were chosen in each plant for the measurement. The middle leaf segments were selected for the analysis, and the process was repeated three times. Finally, the average value was calculated for subsequent analyses.

The photosynthetic parameters, including photosynthetic rate (P_n_), stomatic conductance (G_s_), intercellular CO_2_ concentration (C_i_), and transpiration rate (T_r_), were measured between 9:00 am and 11:00 am on three continuous days using a CIRAS-3 portable photosynthesis system (Lufthansa Technology Group, Beijing, China) under ambient 400 μmol·mol^-1^ CO_2_ concentrations, 1200 μmol m^-2^ s^-1^ photon flux density. The leaf selection was the same as that for the relative chlorophyll content.

The chlorophyll fluorescence parameters were measured by a portable fluorometer chlorophyll fluorometer (DUal-PAM-100, Walz, Effeltrich Germany) on the same day as previously described (Yang et al., 2016), but with minor modifications. The selected leaves were adapted in the dark for 30 min before each measurement. Minimum fluorescence (*F_0_*) was estimated under a low light intensity of 0.1 μmol·m^-2^·s^-1^, whereas the maximum fluorescence (*F_m_*) was determined by a white light-saturating pulse of 6,000 μmol·m^-2^·s^-1^ for 0.8 s. When the fluorescence decreased from the maximum rate of almost *F_0_*, 40 µmol·m^-2^·s^-1^ actinic light was used to induce fluorescence kinetics. The chlorophyll fluorescence parameters, including the maximal efficiency of PSII photochemistry (*F_v_*/*F_m_*), potential photochemical activity (*F_v_*/*F_o_*), the actual efficiency of PSII photochemistry (*Φ_PSII_*), photochemical quenching coefficient (*qP*), and non-photochemical quenching coefficient (*qN*) were calculated. In the Rapid Light Curve window, the photosynthetically active radiation (PAR) was set as 0, 81, 186, 281, 396, 531, 701, 801, 926, and 1076 µmol·m^-2^·s^-1^ at an interval of 20 s. The light response curve was used to obtain the PSII relative electron transfer rate (ETR) and initial quantum yield (α), according to the following formula:


(1) ETR = 0.84 × PAR × ΦPSII/2

### The ultrastructure of chloroplast under a transmission electron microscope

The ultrastructure of leaf chloroplasts under different light qualities on different days (14 d and 56 d) was observed using a TEM as previously described by Oi et al. (2020). Briefly, small leaf segments (approx. 1 mm ×2 mm) were cut from the middle portion of the leaf blades and soaked in 3.0% glutaraldehyde fixative solution for 3 h. The samples were washed four times with 0.1 M phosphate buffer (pH=7.2) and fixed in 1% OsO_4_ dissolved in cacodylate buffer for 2 h at 20 °C. The samples were dehydrated through serial ethanol concentration (30%, 50%, 70%, 80%, 90%, 95%, and 100%) for 15 min in each concentration. After dehydration three times in acetone (15 min each time), the leaf samples were embedded in propylene oxide resin, soaked in an epoxy resin-accelerated mixture, and incubated at 45 °C in the oven for 24 h and thereafter at 60 °C oven for 24 h. The leaf samples were cut transversely into 60 nm thick sections using an ultramicrotome equipped with a diamond knife (EM UC6, Leica, Germany). The leaf sections were stained with lead citrate and uranyl acetate and observed under a transmission electron microscope (Hitachi H-7500, Tokyo, Japan).

### Observation of the stomata

Based on the morphology and photosynthesis efficiency results, mature leaves under different light quality treatments were collected at 28 d for observation using a SEM. The leaf section samples were processed as described by Liu et al. (2021) and observed by a Hitachi S-4700 SEM (Tokyo, Japan).

### Measurements of the mass for the bulbs, leaves, and flowers

The bulbs and leaves at 14 d, 28 d, 42 d, 56 d, and 70 d were collected for measurement of fresh and dry mass by an electronic balance. Fresh bulb samples were weighted and dried in an oven at 80 °C before reweighing until they attained a constant weight. Hierarchical clustering was performed based on their weight using the MeV (Multi Experiment Viewer) tool (https://www.tm4.org/mev.html) (Wang et al., 2020).

### Measurements of the sucrose, glucose, and fructose contents in the bulbs

Bulbs at 0 d, 14 d, 28 d, and 70 d were collected to measure sucrose, glucose, and fructose content. The three components were measured as described by Liu et al. (2021). Differences between groups were assessed by analysis of variance using the SAS software, v. 9.2.

For the sample preparation, bulb scales were ground into powder in liquid nitrogen, and 1.00 g of sample was added to 100 ml of ultrapure water and mixed through ultrasonic shaking for 30 min. The mixture was centrifuged at 16,000 g for 10 min, and the supernatant was filtered through a 0.2 μm filter membrane and diluted 2000 times with diluted water. The contents of the three above sugar were determined by ion chromatograph (DIONEX ICS-3000, Thermo, USA) using a DIONEX ICS-3000 ion chromatography system (Thermo Fisher, Carlsbad, CA, USA), according to the manufacturer’s instructions.

### Total RNA extraction and gene expression analysis

Flower buds and flowers were collected from 0 d to 70 d, while leaf samples were collected from 14 d to 70 d. The samples were immediately frozen in liquid nitrogen and stored at -80 °C until further analysis. The total RNA from the samples was collected, extracted and reverse transcribed to cDNA synthesis according to Wang et al. (2015, 2020) protocol. The primers for amplifying photosynthesis and flowering-related genes were designed by Primer 5.0 (PRIMER Biosoft, San Francisco, CA, USA). Details of the primers are shown in **Table S1**. The *HpEF-1α* and *HpGAPDH2* genes were used as reference genes (Liu et al., 2018). The qRT-PCR reaction system was performed as described by Wang et al. (2015) and Zhu et al. (2018). Each qRT-PCR analysis was performed in triplicate.

## Results

### The light quality affected the plant growth and the onset and duration of flowering of ‘Red Lion’

**Figure 1:**
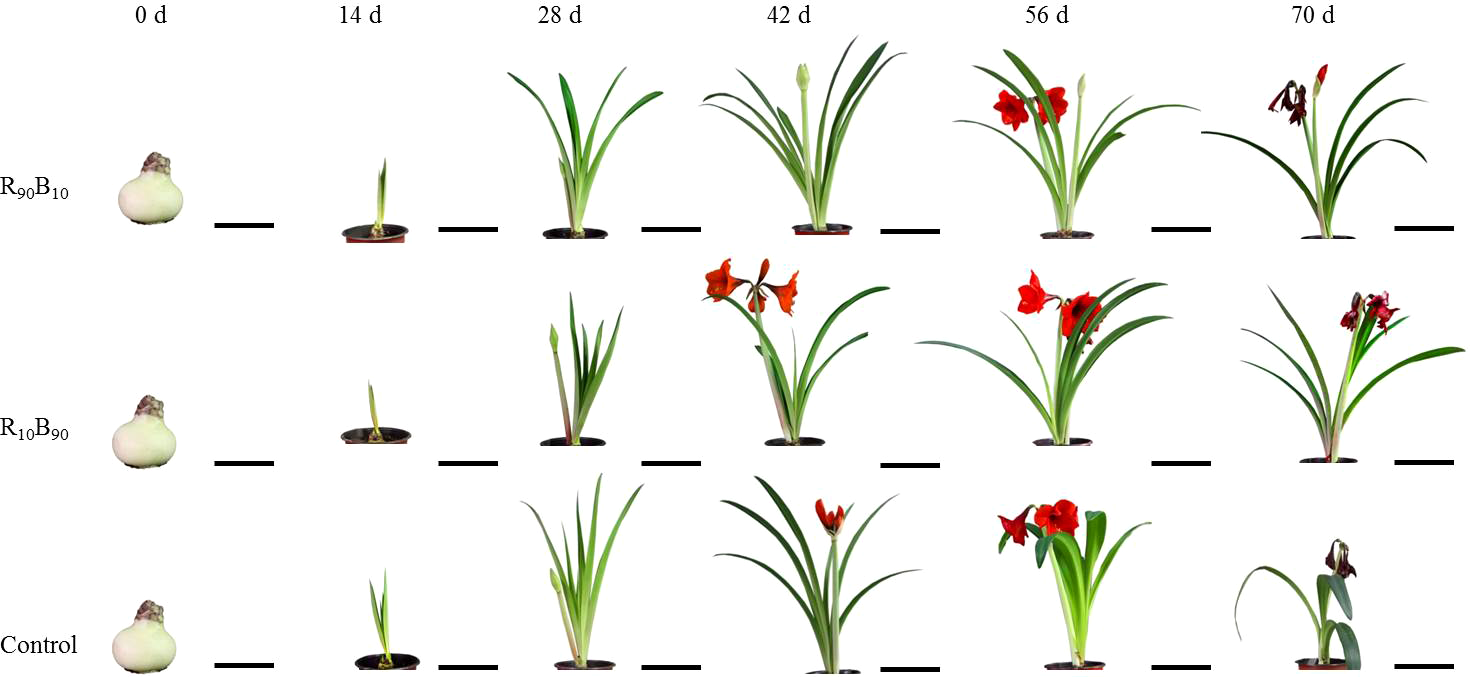
The changes in the morphological characteristics of ‘Red Lion’ at six growth periods under different light quality treatments. R_90_B_10_ and R_10_B_90_ represent combinations of 90% red light and 10% blue light, and 10% red light plus 90% blue light, respectively. The bar value of 0 d and 14 d was 10 cm, while at 28 d - 70 d was 20 cm in.

Compared with that in control and R_90_B_10_ group, at day 14, the leaf growth was slower in the R_10_B_90_ group (**Figure 1**; **Table S2**). Further analysis showed that the leaf length of ‘Red Lion’ R_10_B_90_ group reached 49.72 ± 2.20 cm at 42 d of blooming, shorter than those 55.72 ± 1.44 cm and 52.81 ± 1.26 cm for Red Lion in the R_90_B_10_ and control group, respectively (**Figure 1**; **Table S2**). At 56 d, the leaf length in the R_10_B_90_ group reached 57.25 ± 2.04 cm, shorter than 58.49 ± 2.11 cm and 59.91 ± 1.18 cm for Red Lion in the R_90_B_10_ and control groups, respectively (**Figure 1**; **Table S2**). However, the leaf width at 56 d for Red Lion in the R_10_B_90_ group was 4.98 ± 0.10 cm. In the R_10_B_90_ group, the ratio of length and width of the leaf was 11.51 ± 0.48, implying that the plant was architecturally compact. The leaf length of Red Lion in the R_90_B_10_ group was 62.23 ± 2.64 cm, which was longer than that of Red Lion in the R_10_B_90_ (59.37 ± 2.28 cm) and control (59.89 ± 2.15 cm) groups (**Figure 1**; **Table S2**). The results suggest that R_90_B_10_ promotes leaf growth, including leaf length and width, while the ratio of leaf length and width in R_10_B_90_ is much more proper (**Table S2**). The average length of the longest root and fibrous root and the number of fibrous roots were the longest and highest, respectively, in the R_90_B_10_ and control groups, but the number of roots was fewer in the R_10_B_90_ group (**Table S3**). These root characteristics suggest that R_90_B_10_ promotes plant growth. However, the bulb at 70 d and at 0 d was biggest in the R_10_B_90_ group, moderate in the R_90_B_10_ group, and smallest in the control group (**Table S3**), implying that R_10_B_90_ improved bulb growth.

Regarding the flowering period, flower bud appeared latest in R_90_B_10_ group but earlier in the R_10_B_90_ group (**Figure 1**). Flower opening in the R_10_B_90_ first occurred 2.23 d and 4.26 d earlier than in the control and R_90_B_10_ treatment (**Table 1**). There was a significant difference in the flowering period of the whole plant among the three groups (**Table 1**). The flowering period of the whole plant in group R_10_B_90_ was 18.25 ± 0.48 days, 1.92 d, and 3.45 d longer than those in the control and R_90_B_10_ groups, respectively (**Figure 1**, **Table 1**). The leaf growth results and flowering period analysis implied that a higher red light intensity promoted vegetative growth, especially leaf length, but delayed the time to flowe. In contrast, high blue light promoted flowering, improved the vegetative growth, and prolonged the flowering period of the whole plants (**Figure 1**, **Table 1** and **Table S2**).

**Table 1:** The effect of light quality on flowering period and duration of ‘Red Lion’.

Treatments	Day of first flower opening (d)	The flowering period of a single flower (d)	The flowering duration of the whole plant (d)
R_90_B_10_	48.44 ± 0.71a	5.64 ± 0.20a	14.80 ± 0.20a
R_10_B_90_	44.18 ± 0.61c	4.94 ± 0.34a	18.25 ± 0.48bc
**Control**	46.41 ± 0.41b	5.23 ± 0.22a	16.33 ± 0.33ab

a, b, and c indicate significant differences at the P< 0.05 level. The same was as follows.

### Flowering quality analysis and flower color identification

To investigate the effects of different light quality on the flowering quality, flowering quality-related parameters, including the number of scapes, flowers, and leaves, the scape height, scape and flower diameter, the maximum petal height, the maximum petal width, plant height, crown width, and the ratio of the number of flower to the leaves were analyzed. There were significant differences in the maximum petal length and crown width between the light quality groups. The stalk length, maximum petal length, and crown width were lengthier in the R_90_B_10_ group, reaching 2.66 ± 0.35 cm, 12.23 ± 0.19 cm, and 70.88 ± 3.51 cm, respectively (**Table 2**), implying that R_90_B_10_ promoted both vegetative and reproductive growth. The crown width was narrower for the R_10_B_90_ group (**Table 2**), suggesting the R_10_B_90_ treatment promoted compacted plant architecture formation (**Figure 1**).

**Table 2:** The effect of light quality on the flower quality parameters of ‘Red Lion’.

Treatments	No. of scape	No. of flower	Height of stalk (cm)	Height of scape (cm)
R_90_B_10_	1.60 ± 0.24a	3.40 ± 0.40a	2.66 ± 0.35b	47.92 ± 1.63a
R_10_B_90_	1.80 ± 0.20a	3.40 ± 0.40a	2.54 ± 0.19ab	45.90 ± 1.61a
**Control**	1.60 ± 0.24a	3.00 ± 0.45a	2.34 ± 0.17ab	46.40 ± 0.83a
**Treatments**	Scape diameter (cm)	Flower transverse diameter (cm)	Flower longitudinal diameter (cm)	Length of the maximum petal (cm)
R_90_B_10_	1.68 ± 0.10ab	13.92 ± 0.72a	14.50 ± 1.04a	12.23 ± 0.19a
R_10_B_90_	1.71 ± 0.06a	13.14 ± 0.78a	14.12 ± 0.55a	11.97 ± 0.35b
**Control**	1.58 ± 0.08a	13.34 ± 0.53a	14.10 ± 0.51a	12.20 ± 0.70ab
**Treatments**	Width of maximum petal (cm)	Plant height(cm)	Crown width (cm)	Ratio of No. of flower and leaf
R_90_B_10_	7.63 ± 0.27a	67.30 ± 1.91a	70.88 ± 3.51b	0.87 ± 0.04a
R_10_B_90_	8.03 ± 0.18a	63.64 ± 2.59a	56.00 ± 2.15a	0.89 ± 0.03a
**Control**	7.80 ± 0.10a	64.28 ± 1.77a	63.48 ± 2.82ab	0.89 ± 0.04a

The different letters indicate significant differences at the P < 0.05 level.

**Figure 2:**
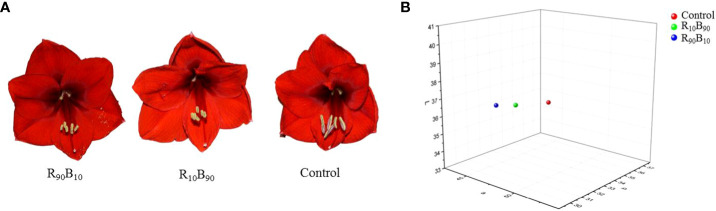
The morphological **(A)** and the distribution of chromatic coordinates of flower color **(B)** under three different light quality treatments.

The chromatic flower color parameters under different light quality treatments were recorded using the CIE L^*^a^*^b^*^ color system (**Table S4**). There was no significant difference in the CIE L^*^a^*^b^*^ c^*^ values between two treatments, but *h^c^* was lower in the two treatment groups (**Table S4**). The flower pigmentation results showed that the flowers in the R_90_B_10_, and R_10_B_90_ groups were dark red and light red, respectively (**Figure 2A**).

Tri-dimensional graphics analysis showed that there was no difference in the color of flowers in the R_90_B_10_ and control group (in the same room), but those in the R_10_B_90_ were in a different room (**Figure 2B**). These results suggested that R_90_B_10_ and R_10_B_90_ treatments all affected the flower color of ‘Red Lion’ (**Figure 2**).

### Photosynthetic measurements and analysis of chlorophyll contents

**Figure 3:**
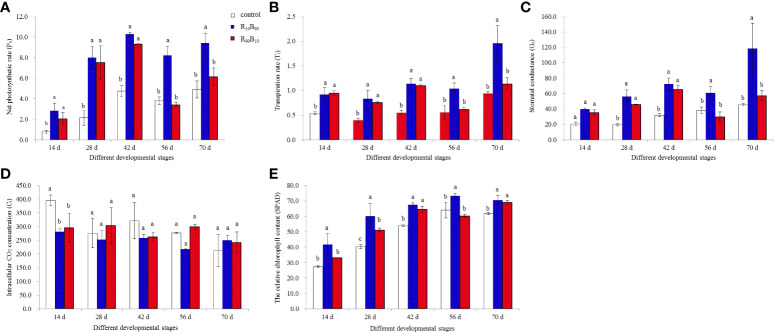
The effect of light quality on the net photosynthetic rate (P_n_) **(A)**, transpiration rate (T_r_) **(B)**, intracellular CO_2_ concentration (C_i_) **(C)**, stomatal conductance (G_s_) **(D)**, and the relative chlorophyll content (SPAD) **(E)** in leaves. Different letters in the same column indicate significant differences at P< 0.05.

The four photosynthetic parameters are shown in **Figure 3**. At 56 d - 70 d, the highest net photosynthetic rate (P_n_) was observed in the R_10_B_90_ treatment, while P_n_ was similar in two treatment groups but were both higher than in control group (**Figure 3A**). At 0 d, 56 d, the lowest P_n_ was observed in the R_90_B_10_ treatment. There was high photosynthesis in mature leaves of plants in the two light quality groups. Transpiration rate (T_r_) was also high in two treatment groups at 14 d, 28 d, and 42 d, relative to the control (**Figure 3B**), and remained high in R_10_B_90_ at 56 d and 70 d. However, the T_r_ in R_90_B_10_ was comparable to the control group at 56 d and 70 (**Figure 3B**). G_s_ trend was similar to that of T_r_, except at 56 d (**Figure 3C**). These findings further indicated that R_10_B_90_ enhanced photosynthesis. The intracellular CO_2_ concentration was lower in the treatment groups than in the control group from 14 d to 42 d, different from P_n_ and T_r_, but it was higher in the R_90_B_10_ group at in the R_10_B_90_ group at 56 d and 70 d (**Figure 3D**). These results suggested that compared with that of white LEDs, red and blue light enhances photosynthesis efficiency in ‘Red Lion’.

The relative chlorophyll content (SPAD) gradually increased with plant growth, except at 56 d for plants in the R_90_B_10_ group, which decreased (**Figure 3E**). Generally, the chlorophyll content was highest for Red lion plants in the R_10_B_90_ group, followed by R_90_B_10_, both of which were higher than plants in the control group (**Figure 3E**).

### Chlorophyll fluorescence measurements

**Figure 4:**
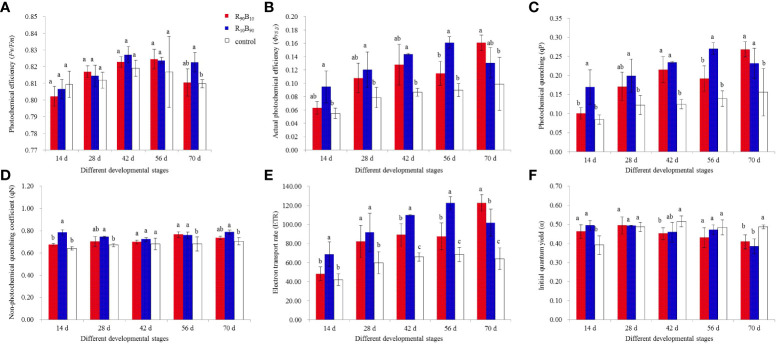
The effect of light quality on photochemical efficiency (*F_v_/F_m_*) **(A)**, photochemical efficiency (*Φ_PSII_*) **(B)**, photochemical quenching (*q_P_*) **(C)**, non-photochemical quenching coefficient (*q_N_*) **(D)**, electron transport rate (ETR) **(E)**, and initial quantum yield (α) **(F)**. Different letters in the same column indicate a significant difference at P< 0.05.

Chlorophyll fluorescence was used to assess plant photosynthesis rate. It also reflects the absorption, transmission, dissipation, and distribution of light energy in photosynthetic systems (Liu et al., 2020). The potential photochemical efficiency (*F_v_/F_m_*) increased at 14 d - 42 d and decreased at 56 d - 72 d in the treatment and control group over for the whole developmental stages and peaked at 0.827 ± 0.005, 0.825 ± 0.006, and 0.819 ± 0.005, at 42 d, 56 d, and 42 d, respectively, in R_10_B_90_, R_90_B_10_, and control, respectively (**Figure 4A**; **Table S5**). Generally, *F_v_/F_m_* was higher in the leaves of Red lion in the R_10_B_90_ group than in the R_90_B_10_ groups, but both were higher than the control group (**Figure 4A**). The photosystem II reaction center was stronger under higher blue light. The photochemical efficiency (*Φ_PSII_*) was higher in the R_10_B_90_ than in the R_90_B_10_ group from 14 d to 56 d, and it was sharply increased at 70 d in the R_90_B_10_ group, peaking at 0.161 ± 0.011 (**Figure 4B**; **Table S5**). Generally, blue light improved the photochemical efficiency in the leaf in most of the developmental stages.

The trend in the photochemical quenching (*qP*) was similar to that of *Φ_PSII_*, which was significantly higher in the R_10_B_90_ treatment group from 14 d to 56 d (**Figure 4C**). Meanwhile, the maximum *qP* (0.268 ± 0.02) in the R_90_B_10_ treatment group occurred at 70 d (**Figure 4C**; **Table S5**). The trend in the non-photochemical quenching coefficient (*qN*) was similar to that of *qP* (**Figure 4D**). In contrast, *qP*, *qN* was highest in the R_10_B_90_ group at 70 d, reaching a maximum of 0.787 ± 0.018 (**Figure 4D**; **Table S5**). In general, higher blue light (R_10_B_90_) enhanced *qN*. Meanwhile, the electron transfer rate (ETR) was significantly higher in the treatment groups than in control (**Figure 4E**). The initial quantum yield (α) was higher in the R_10_B_90_ group than in the control group but was lower in the R_90_B_10_ group than the control group at 14 d (**Figure 4F**). Chlorophyll fluorescence rates suggested that an optimal combination of blue and red light enhanced ‘Red Lion’ growth.

### The chloroplast ultrastructure

**Figure 5:**
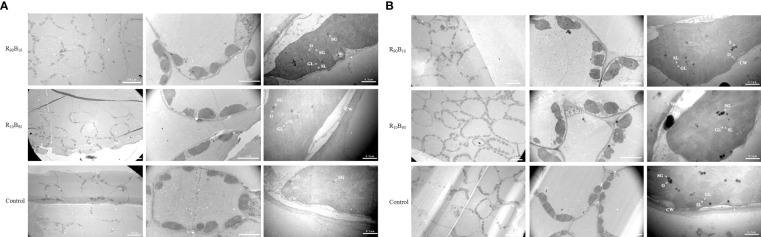
The effect of light quality on the chloroplast ultrastructure of ‘Red Lion’ under TEM at 14 d **(A)** and 56 d **(B)**. CW, GL, O, SG, and SL represent cell walls, grana lamella, osmiophilic granule, starch granule, and stroma lamella, respectively.

The chloroplast structure is very important for plant growth and photosynthesis. The leaves at the initial developmental stage and flower blooming stage were selected for chloroplast ultrastructure observation. At 14 d, chloroplast appeared long ellipse or shuttle across the treatments and the control group, and the chloroplast was closed to the cell membrane, suggestive of good chloroplast development across the treatments. Meanwhile, immature starch granules were observed, but grana and stroma lamella were not clearly observed (**Figure 5A**). At 56 d, the chloroplast structure developed into ellipse type, and all the structures had well developed (**Figure 5B**). In the control group, the starch granules were closely packed and small; and grana lamella and osmiophilic granule were apparent. The starch and osmiophilic granules were smaller in the R_10_B_90_ group than in the other two groups, but the starch granules were large in control group. The grana thylakoid density was higher in the R_10_B_90,_ and grana lamella was well packed (**Figure 5B**), suggesting all of which enhanced photosynthesis in Red Lion. The number and volume of starch granules were all larger in the R_90_B_10_ treatment than in the other two treatments. The grana lamella was also well packed, and only a few osmiophilic granules were present (**Figure 5B**). Overall, the two light treatments did not destroy the ultrastructure of chloroplast, but the chloroplast development in the R_10_B_90_ and R_90_B_10_ groups was better than in the control treatment.

The chloroplast grew more during leaf development (from 14 d to 56 d) and the number of chloroplast reached to 2.5, 9.33, and 3.5, respectively, in the R_90_B_10_, R_10_B_90_, and control groups (**Figure 5**; **Table 3**). The number of chloroplasts was highest in the R_10_B_90_ group (**Table 3**), implying that higher blue light intensity increased the numbers of chloroplasts and, thus, high photosynthesis.

**Table 3:** The effect of light quality on the numbers of chloroplast in the leaves of ‘Red Lion’ at 14 d and 56 d.

Treatments	Different developmental stages	
	14 d	56 d
R_90_B_10_	11.33 ± 3.54a	13.83 ± 4.17a
R_10_B_90_	10.00 ± 2.08a	19.33 ± 5.12b
Control	8.83 ± 0.90a	12.33 ± 2.28a

The different letters indicate significant differences at the P < 0.05 level.

### Stomatal analysis using a scanning electron microscope

The stomatal movement affects the photosynthetic efficiency of plants. The density and size of the stomata were significantly different under the light treatments (**Figure S1**; **Table 4**). Particularly, the stomatal density was highest in the R_10_B_90_ group, peaking at 65.63 ± 4.13 per mm^2^, which was 2.60 times more than in the R_90_B_10_ group (**Table 4**). The stomata were also the largest in the R_90_B_10_ group, followed by the control group and the R_10_B_90_ group. In contrast, the stomata width was the smallest in the R_90_B_10_ group, reaching around 14.3 ± 1.33 µm (**Figure S1**; **Table 4**). The stomatal area in the R_10_B_90_ and the control group was larger than in the R_90_B_10_ group. The stomata always widely opened in the R_10_B_90_ and R_90_B_10_ than in the control group (**Figure S1**), which enhanced photosynthesis.

**Table 4:** The effect of light quality on stomatal characteristics of ‘Red Lion’ leaf at 28 d.

Treatments	Density of stomata (No./mm^2^)	Length of stomata (µm)	Width of stomata (µm)	Area of stomata (μm^2^)
R_90_B_10_	25.22 ± 2.67a	48.55 ± 0.86b	14.3 ± 1.33a	695.73 ± 72.21a
R_10_B_90_	65.63 ± 4.13b	44.16 ± 0.97a	18.1 ± 2.03b	803.61 ± 97.72b
**Control**	54.76 ± 2.33b	46.19 ± 2.93ab	17.4 ± 2.66b	805.29 ± 142.95c

The different letters indicate significant differences at the P < 0.05 level.

### Changes in the fresh and dry weight of bulbs, leaves, and flowers

**Figure 6:**
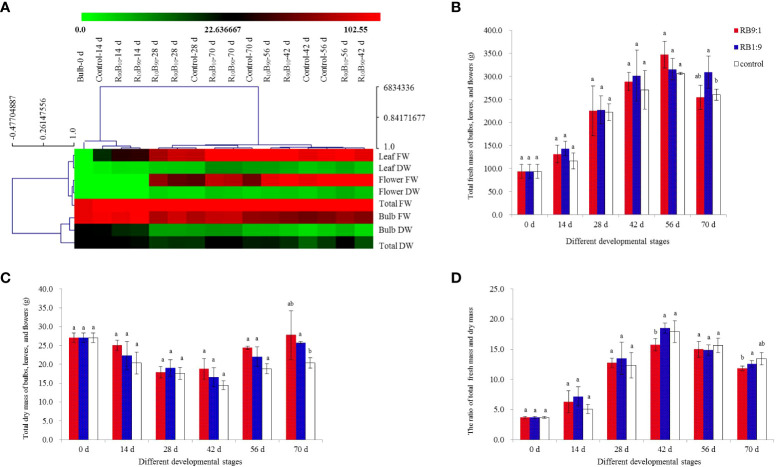
Hierarchical clustering analysis (HCA) for the effect of light quality on the fresh and dry mass of red lion bulb, leaf, flower, and the sum of three. **(A)** total fresh mass **(B)**, dry mass **(C)**, and the ratio of fresh and dry mass **(D)**. Different letters indicate significant differences at P< 0.05.

Fresh and dry weight of bulbs, leaves, flowers, and the total sum of three organs were determined to investigate the effects of different light qualities on plant carbohydrate accumulation and consumption, respectively. The hierarchical clustering analysis (HCA) of the fresh and dry weight of different organs showed that the treatments could not be clustered together at 28 d and 42 d (**Figure 6A**) during the rapid flower bud development stage and flowering stage, respectively (**Figures 1**, **6A**). It was deduced that the fresh and dry was significantly different in these two stages. Meanwhile, HCA analysis further revealed that the trends in the dry and fresh mass of the mentioned organs were the same at 70 d and 28 d (**Figure 6A**).

The total fresh mass of ‘Red Lion’ in different treatment groups increased continuously from 14 d to 56 d and then decreased at 70 d, and the mass was higher in the R_90_B_10_ and R_10_B_90_ groups than in the control group (**Figure 6B**). Meanwhile, the total fresh mass of total three organs was highest in the R_10_B_90_ group. The total dry weight of the Red Lion decreased from 0 d - 42 d, across the three treatments and was lowest at the flowering stage (42 d), but it increased from 56 d - 72 d (**Figure 6C**). In general, the dry mass of the R_90_B_10_ and R_10_B_90_ groups was higher than the control group, though statistically insignificant across the three groups (**Figure 6C**), indicating a high accumulation of dry matter. The lower fresh and dry weight ratio in the treatment groups at 56 d and 72 d was also suggestive of high carbohydrate accumulation (**Figure 6D**).

The fresh mass of bulbs decreased with the reproductive growth period. The dry mass of bulbs and the fresh and dry mass ratio showed similar patterns as those of the total biomass of bulbs, leaves, and flowers (**Figure S2**). From 14 d to 42 d, the fresh weight of bulbs across the three treatment groups decreased sharply and was lowest in the control group at 42 d and remained 57.69 ± 6.69 g in this group. The highest weight was reached at 70 d, reaching 84.01 ± 4.89 g in the R_10_B_90_ group (**Figure S2A**). These results suggested that flowering needs carbohydrates, and the larger bulk of the carbohydrates are accumulated at 56 d - 70 d. The total dry mass was highest for the bulbs, leaves, and flowers; and the change in the mass of bulbs affected the growth and flowering of ‘Red Lion.’ Collectively, the fresh weight of bulbs in the R_90_B_10_ and R_10_B_90_ group remained high in the whole developmental stages, while the dry mass of bulbs in the two treatment groups was high at 28 d - 70 d (**Figure S2B**). For the decrease pattern of dry weight of bulbs, it is thought that flowering needed nutrients from the bulbs, but the need for carbohydrates was lower in the R_10_B_90_ than in the other two treatments (**Figure S2B**). The fresh and dry weight ratio of the bulbs further indicated that plant growth was very active at 42 d, and the dry weight was higher in R_10_B_90_ and R_90_B_10_ groups at 56 d - 70 d (**Figure S2C**).

The fresh and dry weights of leaves in the three treatments all increased and compared with those in the control group, and they were high in R_10_B_90_ and R_90_B_10_ groups at almost all the developmental stages (**Figures S2D–S2F**). Generally, a combination of red and blue light improved leaf growth and accumulation of carbohydrates in the leaves. At 56 d and 70 d, the fresh weight of leaf in R_10_B_90_ were significantly higher than in the other two treatments, reaching 128.20 ± 9.49 g and 146.10 ± 11.66 g, respectively (**Figure S2D**; **Table S5**). Meanwhile, the dry weight of leaves at 56 d was highest in R_10_B_90_, moderate in R_90_B_10_, and lowest in the control group (**Figure S2E**). Between 14 d to 42 d, the dry weight of leaves was highest in the R_90_B_10_ group. The ratio of the fresh and dry weight of leaves was high in R_10_B_90_ at 42 d and the control group at 56 d (**Figure S2F**). In general, blue and red light improved the leaf growth, increased the fresh and dry mass, and promoted the accumulation of carbohydrates in ‘Red Lion’ leaf. Given the higher mass accumulation of bulbs at 56 d (**Figures S2A, B**), it was thought carbohydrates are transferred to bulbs from 56 d.

Flower bud appeared at 28 d. It was found that fresh and dry weights of flowers in the three groups increased from 28 d - 56 d, and decreased at 70 d (**Figures S2G**, **S2H**). At 56 d, the highest fresh and dry flower mass was observed in the R_90_B_10_ group, followed by R_10_B_90_, and control (**Figure S2H**). The probable reason is that the earlier flowering in the R_10_B_90_ group corrected with earlier weight accumulation from 28 d to 42 d, which then decreased during the flower-withering period from 56 d to 70 d. Meanwhile, the ratio of fresh and dry weight of flowers has not significantly different among three groups (**Figure S2I**).

### Glucose, fructose, and sucrose accumulation in the bulbs of Red Lion

**Figure 7:**
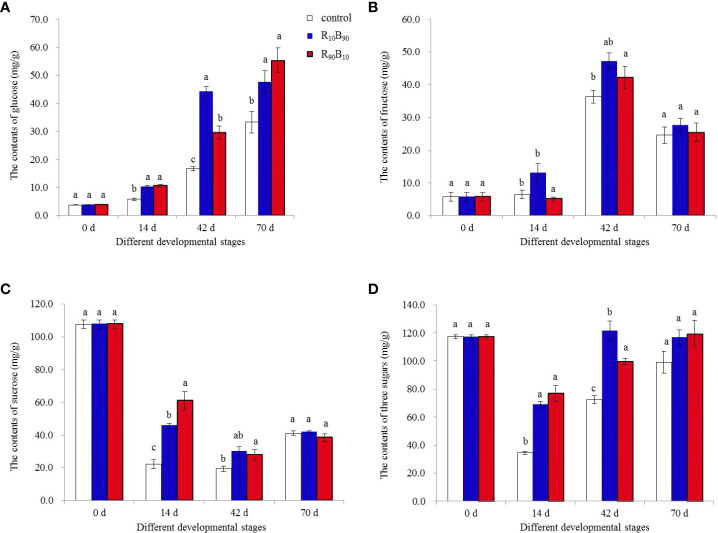
The effect of light quality on the glucose **(A)**, fructose **(B)**, sucrose **(C)**, and total sugars **(D)** content at important developmental stages at 0 d, 14 d, 42 d, and 70 d. Different letters in the same column indicate a significant difference at P< 0.05.

Glucose, fructose, and sucrose contents in the Red lion bulbs were measured at 0 d, 14 d, 42 d, and 70 d over the growth, flowering, and fresh and dry mass changes. The glucose content increased in the three groups, peaking at xyz. In contrast, the fructose content decreased (**Figures 7A, B**). The highest glucose content at 42 d was in the R_10_B_90_ treatment, which corresponded with the rapid growth of other plant parts (**Figure 1**). Compared with the R_10_B_90_ and the control group, the fructose content was highest in R_10_B_90_ treatment at 42 d. The sucrose content decreased and was high in the R_90_B_10_ treatment at 14 d (**Figure 7C**). It was deduced that sucrose participates in the flowering of Red lion. Therefore, the high concentration of non-structural carbohydrates, including glucose, fructose, and sucrose at 42 d in R_10_B_90_ might be responsible for the rapid flowering of the Red lion. The contents of the three sugars decreased rapidly before 14 d and then increased between 42 d and 70 d (**Figure 7C**). These results indicated that blue and red light promoted the accumulation of non-structural carbohydrates (glucose, fructose, and sucrose) in the red lion (**Figure 7D**).

### Expression analysis of chlorophyll biosynthesis and flowering-related genes

**Figure 8:**
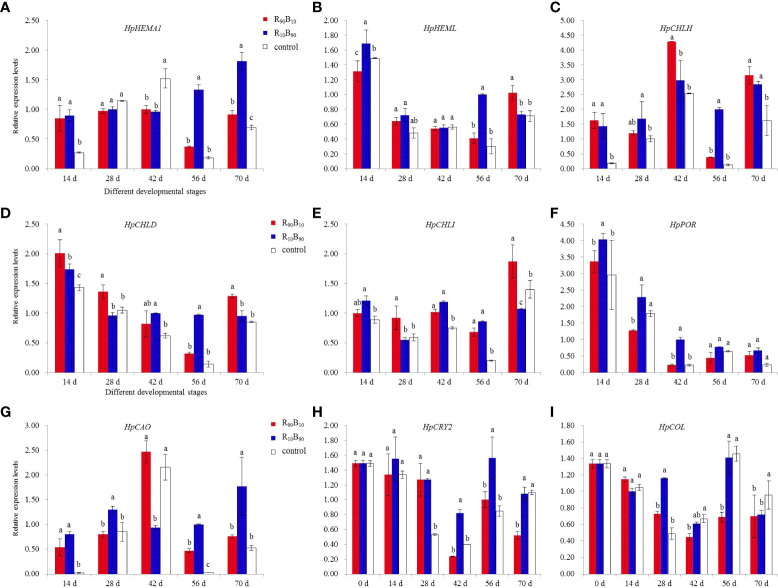
The effect of light quality on expression analysis for seven genes related to chlorophyll biosynthesis and two genes related to flowering time of H. ybrid. **(A–I)** was corresponded to the expression profile of HpHEMA1, HpHEML, HpCHLH, HpCHLD, HpCHLI, HpPOR, HpCAO, HpCRY2, and HpCOL, respectively. Different letters in the same column indicate significant differences at P < 0.05.

Chlorophyll is an indispensable component for photosynthesis. The present study investigated the effect of red and white light on the expression of seven chlorophyll biosynthesis-related genes. The expression of the *HpHEMA1* gene at 14 d, 42 d, 56 d, and 70 d was inconsistent across the three groups. However, the gene was overexpressed in the R_10_B_90_ group at 56 d and 70 d (**Figure 8A**). The expression of the *HpHEML* gene, which regulates 5-aminolaevulinic acid synthesis, was generally overexpressed in the two light treatments than in the control group. The expression was highest in the R_10_B_90_ group at 14 d. The gene was also overexpressed at 56 d and 70 d in two treatment groups (**Figure 8B**). The expression of *HpCHLH/D/I* was significantly high in the two treatment groups at almost all the developmental stages (**Figures 8C–E**). The expression of *HpPOR*, which participates in the divinyl protochlorophyllide *a* to divinyl chlorophyllide synthesis, was also overexpressed in the two light treatment groups (**Figure 8F**). The expression of *HpCAO*, chlorophyll an oxygenase gene, was very high in R_10_B_90_ group, and the lowest expression in this group was observed at 42 d (**Figure 8G**). In general, high blue light intensity promoted the expression of chlorophyll synthesis genes. High chlorophyll content on its part enhanced the photosynthesis rate in Red Lion.

The blue-white light intensity affected the expression of photoreceptor and flowering related genes. For instance, blue light increased the expression of *PHYA*, *CRY2*, and *CONSTANTS* (*CO*). The expression of the *HpCRY2* gene also varied with the flowering gene. *CONSTANTS-LIKE* and *HpCOL* genes were selected for gene expression analysis at the flower budding and across the whole developmental stages. Before flowering, the expression of *HpCRY2* and *HpCOL* was higher at 28 d both in R_10_B_90_ and R_90_B_10_ treatments, while *HpCRY2* was strongly expressed at the flowering period from 42 d to 56 d (**Figures 8H, I**). The high expression of *HpCRY2* and *HpCOL* at 28 d extended the flowering time. Meanwhile, blue light could promote the expression of *HpCRY2* (perceived blue light), which regulates flower bud formation. In the leaf, *HpCRY2* was highly expressed in R_10_B_90_ and R_90_B_10_ treatment at 28 d, and 56 d, respectively (**Figure S3**). It was deduced that blue light induced *HpCRY2* expression in leaf and flower buds, and high expression of *HpCRY2* promoted flowering of ‘Red Lion’.

## Discussion

LED lights are more effective than fluorescent lamp lights in promoting plant vegetative growth, and varying the intensity and duration of red and blue light affects shoot elongation and flowering (Li et al., 2012; Gautam et al., 2015). Red and blue lights are often used to improve the yields and quality of crops cultivated in the greenhouse (Kaiser et al., 2019). LEDs with R_90_B_10_, and R_10_B_90_ combinations and white LEDs were designed to investigate the effects of red and white light intensities on the vegetative and reproductive growth of ‘Red Lion’. We found that R_10_B_90_ and R_90_B_10_ significantly affected the vegetative growth and flower quality of ‘Red Lion’ under forcing culture.

### R_90_B_10_ light combination promoted vegetative growth of ‘Red Lion’ under forcing culture

Optimal blue light increases the total biomass, yield, and the number of tomato fruits (Kaiser et al., 2019), while red light increases the fresh weights of shoots, leaves, and the height of preilla plant (Nguyen and Oh, 2021). High red light intensity (R_90_B_10_) induces vegetative growth in lettuce and perilla, particularly the leaf length, scape, and plant height (Naznin et al., 2019; Nguyen and Oh, 2021). Red LEDs improved fresh weight of perilla roots (Nguyen and Oh, 2021), while R_90_B_10_ promotes fibrous root growth of ‘Red Lion’. It was deduced that red light affected plant root development. Nevertheless, higher blue light (R_10_B_90_) inhibited leaf growth but promoted flower biomass and total biomass growth, lower bud growth, and flowering at 28 d and 42 d, and at 70 d, respectively. R_10_B_90_ promotes leaf widening and repressed an increase in crown width of ‘Red Lion’. This implies that R_10_B_90_ also promotes compacted plant formation of ‘Red Lion’. Red light reduced shoot elongation and also resulted in more compact plants in *Petunia* × *hybrid* (Gautam et al., 2015), indicating that 1/10 red light quality affected the growth of ‘Red Lion’. The present study revealed that red and blue light combination might promote optimal ‘Red Lion’ growth. A combination of blue and red light has been used in closed plant production systems (Hogewoning et al., 2010).

### R_10_B_90_ light combination promoted photosynthesis and chlorophyll synthesis in ‘Red Lion’ under induced condition

Plants are often exposed to rapidly alternating light intensity and quality. Red and blue light affect leaf photosynthesis (Zhang et al., 2019). The chlorophyll *a*, chlorophyll *b*, and total chlorophyll content of lettuce and kale increased under 91%R + 9%B and 95%R + 5% B light, respectively (Naznin et al., 2019). R_10_B_90_ also increased chlorophyll content and photosynthesis (**Figure 3**). Low red light, in combination with blue light (R_10_B_90_), promotes chlorophyll synthesis and the photosynthetic capacity of ‘Red Lion’, and P_n_ reached to 9.86, which was two times higher than in the control group. In vegetables, there is a positive correlation between the total chlorophyll content and the blue light intensity (Naznin et al., 2019). In rice, compared with blue light, red light decreased the chlorophyll content by 45% (Hamdani et al., 2019), consistent with our results. R_70_B_30_ treatment, but not B_100_, increased the secretion of chlorophyll *a* + b (Zhang et al., 2019). It was further suggested that blue light intensity affects chlorophyll synthesis.

The chlorophyll biosynthesis process comprises 15 reactions catalyzed by 27 enzymes (Beale, 2005). Glutamyl-tRNA reductase (*HEMA*) is the primary enzyme that regulates chlorophyll synthesis, while the glutamate-1-semialdehyde aminomutase 1 (*HEML*) gene codes for an important enzyme that catalyzes 5 aminolevulinic acid synthesis. At 56 d, the leaf well developed (**Figure 3**). The chlorophyll content and P_n_ were high in the R_10_B_90_ group, and the expression levels of *HpHEMA* and *HpHEML* were all highest, compared with those in the R_90_B_10_ group and control group (**Figures 3**, **7**). Magnesium chelatase is another key enzyme in the process of chlorophyll synthesis, which catalyzes the insertion of Mg^2+^ into protoporphyrin IX to form magnesium protoporphyrin IX, composed of three subunits, *CHLH*, *CHLD*, and *CHLI* (Moradi and Ismail, 2007). The expression levels of three genes were also high at 56 d in the R_10_B_90_ group (**Figures 3**, **7**). The protochlorophyllide oxidoreductase (*POR*) gene regulates divinyl protochlorophyllide *a* synthesis, and it was highly expressed in the leaves at 14 d, 28 d, and 32 d in the R_10_B_90_ group. Chlorophyll oxygenase (*CAO*) is an important enzyme in the chlorophyll cycle (Wang and Grimm, 2021), and it was also highly expressed in leaf in the R_10_B_90_ group. Thus, a combination of blue and red light (R_10_B_90_) significantly increased the expression of the above seven genes in well-developed leaves of ‘Red Lion’. High expression of these genes induced chlorophyll synthesis and the corresponding photosynthesis. Blue light also increases the total chlorophyll contents in the seedlings of Chinese cabbage (Li et al., 2012).

The photosynthetic rate in leaves corresponded with the chlorophyll content and growth rate of ‘Red Lion.’ The net photosynthetic rate at ambient CO_2_ and stomatal conductance were highest in the R_10_B_90_ group with high chlorophyll content. Meanwhile, the stomata density and the stomatal opening degree were also high in the R_10_B_90_ group. Previous studies have shown that blue light promotes stomatal development, increases stomatal density, and promotes stomatal opening. High red light intensity and low red light intensity increase the number but reduce the size of stomata in chrysanthemum seedlings (Ramalho et al., 2002; Kim et al., 2004), consistent with our findings. High blue light, in combination with red light, promotes stomatal opening and conductance (Shimazaki et al., 2007). Thus, blue and red light combinations enhance photosynthesis of ‘Red Lion’.

Increasing the proportion of blue light during the leaf growth enhances the photosynthetic capacity of cucumbers and tomatoes (Hogewoning et al., 2010; Kaiser et al., 2019). Chlorophyll fluorescence parameters showed that except for the initial quantum yield (α), R_10_B_90_ increased the maximum quantum efficiency of photosystem II photochemistry (*F_v_/F_m_*), photosystem II quantum yield (Φ_PSII_), photochemical quenching (qP), non-photochemical quenching (qN), and electron transfer rate (ETR). Blue light increases transpiration rate and the photochemical efficiency (*F_v_/F_m_*) in perilla (Nguyen and Oh, 2021).Likewise, high blue light in combination with high or low red light intensity increased *F_v_/F_m_*, qP, and ETR in *P. ahipa* (Ramalho et al., 2002). These results suggested that optimal ratios of blue and red light intensities improve the photochemical efficiency of photosystem II and electron transport and, thus, photosynthesis. Meanwhile, the expression of blue-light receptor gene *HpCRY2* increased under the R_10_B_90_ group with high blue light combination.

### Red and blue light affected the accumulation of physiological molecules and flowering time of ‘Red Lion’

Total biomass analysis showed that both fresh and dry mass was higher in the R_90_B_10_ group than in the R_10_B_90_ group, but both higher than the control from 42 d - 70 d, which corresponded with the growth of reproductive structures. R_10_B_90_ promoted the accumulation of fresh, dry biomass and the total biomass, consistent with previous studies (Kaiser et al., 2019; Naznin et al., 2019). A combination of blue and red light promotes reproductive growth (Li et al., 2012). Optimal blue light increases fruit yields (Kaiser et al., 2019). R_10_B_90_ and R_90_B_10_ promoted vegetative growth but not an increase in the dry mass. It was deduced that flowering consumed carbohydrates, and given the different flowering periods in the three treatments. Thus, the total biomass in the latter stages varies. The lower dry mass in the R_10_B_90_ group on 14 d was caused by its early flowering.

Non-structural carbohydrates as energy substrates and sugar signaling trigger flowering (Liu et al., 2021). We found that compared to the control group, glucose, fructose, and sucrose content were significantly high in the R_10_B_90_ and R_90_B_10_ groups. In related research, red light significantly increased the sucrose and soluble sugar contents (Li et al., 2012), consistent with our results. It was deduced that higher red light intensity promoted sucrose accumulation. The higher glucose and fructose contents might have contributed to ‘earlier Red Lion’ flowering. *CO* is a key gene that regulates the flowering time in many plants (Wang, 2019; Liu et al., 2021). Overexpression of *Vigna radiata COL2* gene (*VrCOL2*) accelerated flowering in *Arabidopsis* under short-day conditions (Liu et al., 2021). High expression of *CO* affected the expression levels of CAPRICE family genes to achieve early flowering (Wada and Tominaga-Wada, 2015). High expression of *HpCOL* gene in R_10_B_90_ promoted early flowering of ‘Red Lion’(**Figures 1**, **8I**). *HpCRY2* was highly expressed in the flower bud and flowers in the R_10_B_90_ group. Light quality can affect the beginning of the flowering of plants. Previous studies have shown that rose, *chrysanthemum*, *petunia*, and *Arabidopsis* flower early under blue light than white light (Masuda and Takamiya, 2004; Abidi et al., 2013; Fan et al., 2014). Compared with white light, blue plus red light lengthens the flowering duration of Chinese cabbage (Li et al., 2012). On the other hand, blue light enhances pigmentation (Naznin et al., 2019), and the two lights brighten the flower color. It can be concluded that higher blue light intensity promotes early flowering and prolongs the flowering period of ‘Red Lion’.

**Figure 9:**
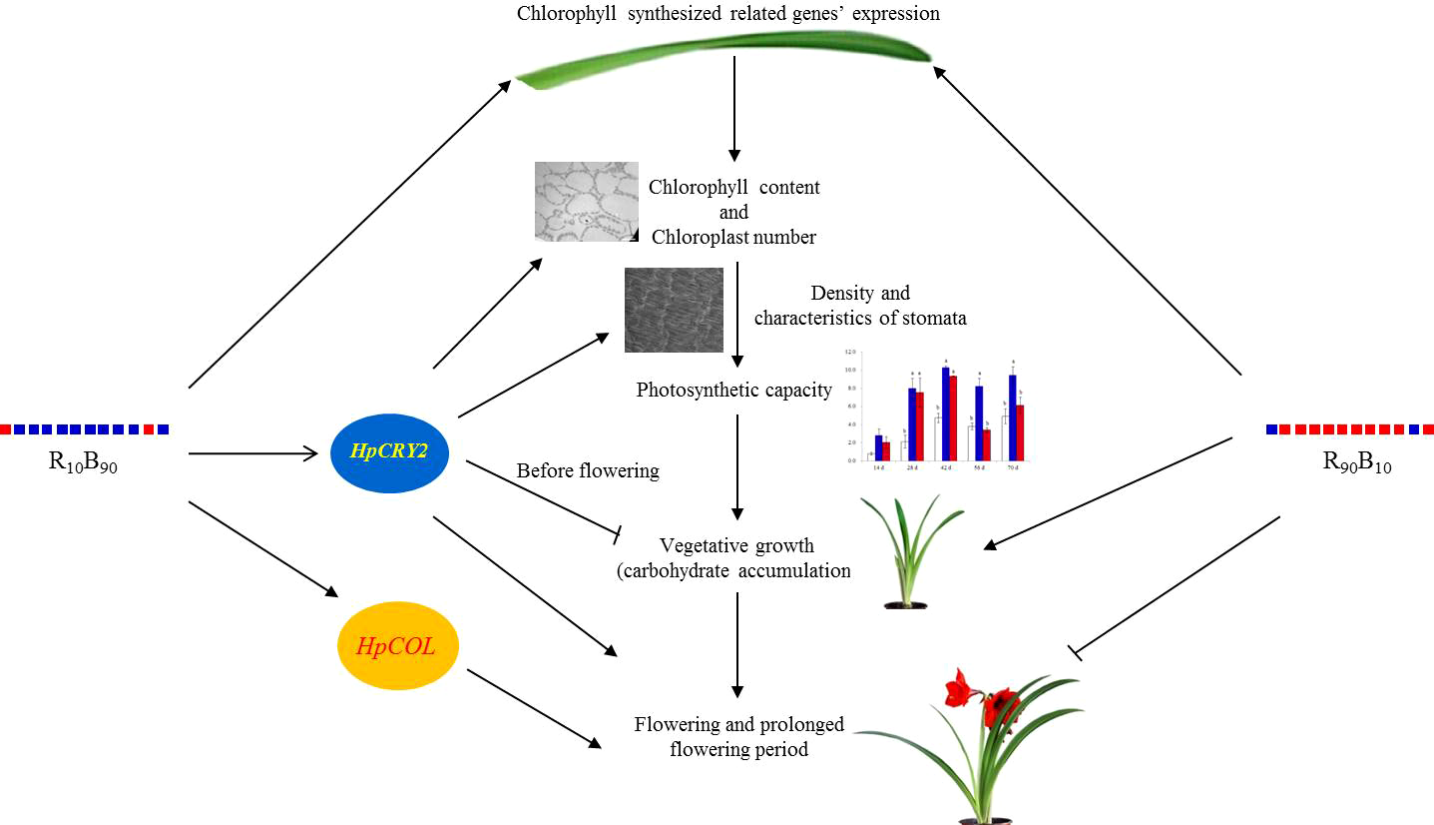
The probable mechanism of how blue and red light combination regulated vegetative and reproductive growth of ‘Red Lion’ under forcing-culture.

In the present study, based on the growth and photosynthesis, contents of non-structural carbohydrates, and gene expression investigation, it was deduced that R_10_B_90_ promoted early flowering while R_90_B_10_ promoted both vegetable and reproductive growth and delayed flowering. However, both R_10_B_90_ and R_90_B_10_ promote chlorophyll synthesis by inducing the expression of related genes (**Figure 9**). High chlorophyll contents enhance photosynthesis rate by increasing light capturing and transfer efficiency, with high carbohydrates, such as glucose, fructose, and sucrose being the end products (**Figure 9**). R_10_B_90_ promoted early flowering by inducing the expression of *HpCOL* and *HpCRY2.* The expression of the two genes also increased the fructose and sucrose contents in bulbs and prolonged the flowering period (**Figure 9**). Meanwhile, R_90_B_10_ induced early flowering and shorted the flowering period (**Figure 9**).

## Conclusion

Suitable light quality is essential for plant growth and development. R_10_B_90_ and R_90_B_10_ induced chlorophyll synthesis and increased the photosynthesis rate. Higher blue light and low (1/10) red light intensity (R_10_B_90_) promoted early flowering and prolonged the flowering period of ‘Red Lion’ under forcing culture. Moreover, R_10_B_90_ promoted the accumulation of non-structural carbohydrates and plant compaction. The higher red light and low blue light (with 1/10) intensity (R_90_B_10_) promoted vegetative and reproductive growth and delayed flowering. However, R_90_B_10_ shortened the flowering period. Both R_10_B_90_ and R_90_B_10_ improved flower color by increasing the production of the flower color pigment.

## Data availability statement

The original contributions presented in the study are included in the article/**Supplementary Material**. Further inquiries can be directed to the corresponding authors.

## Author contributions

SW designed the experiments, drafted the manuscript and analyzed experimental data. XNL performed the experiments. XNL, JX, and XR participated in some data analysis. SW, YZ and XZ supervised the project. The authors take responsibility for all aspects of the reliability and freedom from bias of the data presented and their interpretation. All authors contributed to the article and approved the submitted version.

## Funding

The work was supported by China Agriculture Research System (CARS-21), China Association for Science and Technology Foundation for Young Scholars (2015QRNC001), and the Agricultural Science and Technology Innovation Program (ASTIP) of the Chinese Academy of Agricultural Sciences (CAAS-ASTIPIVFCAAS). The funding bodies have no role in the study design, data collection and analysis, decision to publish, or preparation of the manuscript.

## Acknowledgments

We thank mogoedit for its linguistic assistance during the preparation of this manuscript.

## Conflict of interest

The authors declare that the research was conducted in the absence of any commercial or financial relationships that could be construed as a potential conflict of interest.

## Publisher’s note

All claims expressed in this article are solely those of the authors and do not necessarily represent those of their affiliated organizations, or those of the publisher, the editors and the reviewers. Any product that may be evaluated in this article, or claim that may be made by its manufacturer, is not guaranteed or endorsed by the publisher.

## Supplementary material

Supplementary Figure 1 The effect of different light qualities on the stomatal morphology of ‘Red Lion’ leaves at 28 d under 200 × and 2000 × magnification.

Supplementary Figure 2 The changes in fresh and dry mass and the ratio of the fresh and dry mass of bulb **(A)**, leaf **(B)**, and flower **(C)** under different blue and red light intensities. Different letters indicate significant differences at P< 0.05.

Supplementary Figure 3 The effect of different light qualities on the expression of the *HpCRY2* gene in leaves. Different letters indicate a significant difference at P< 0.05.
