# Supplementary figures and images for: The red/blue light ratios from light-emitting diodes affect growth and flower quality of Hippeastrum hybridum ‘Red Lion’
Source: Front Plant Sci. 2022 Dec 1;13:1048770. doi: 10.3389/fpls.2022.1048770 (PMC9751929; doi:10.3389/fpls.2022.1048770)

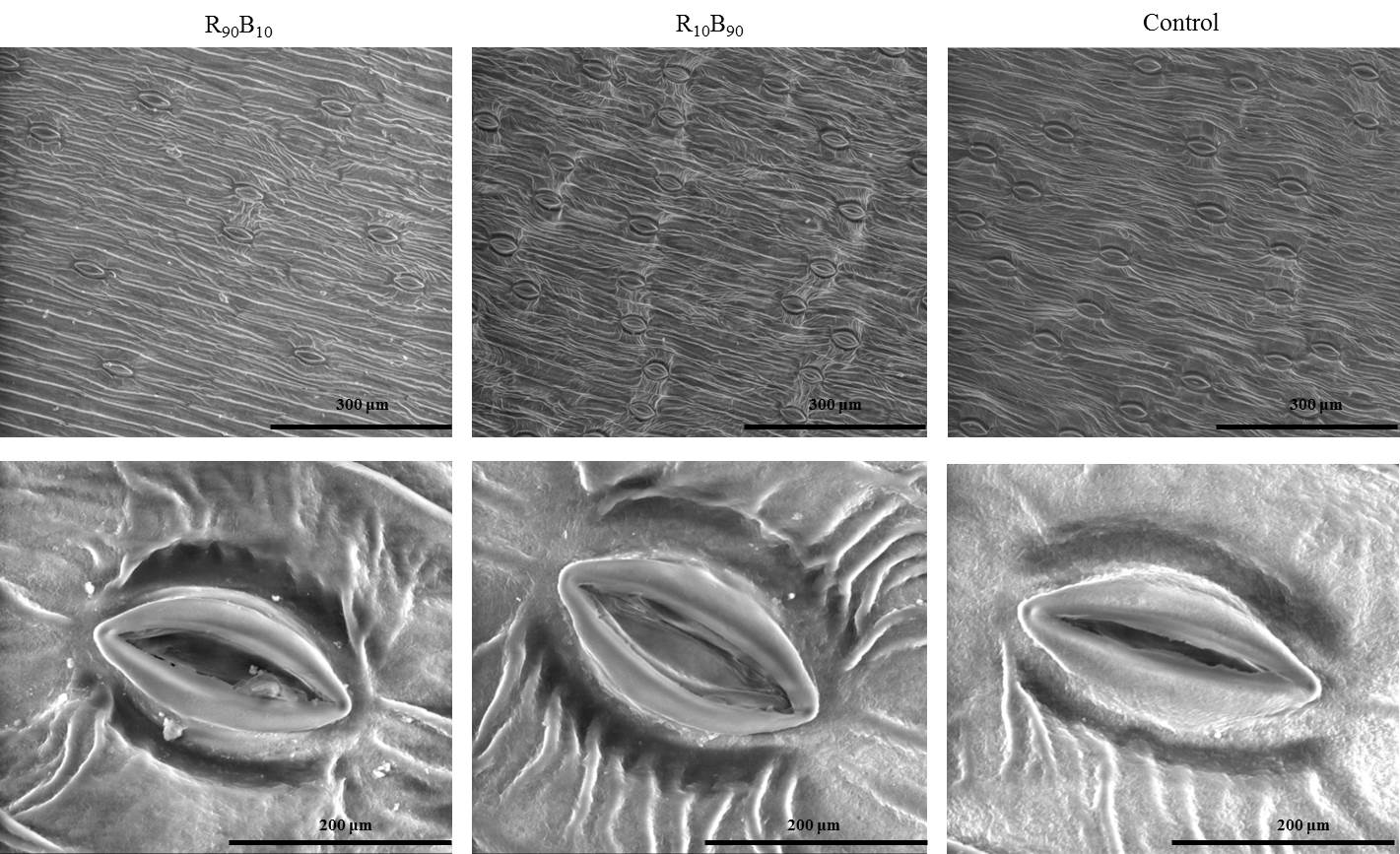

Supplement: Supplementary Figure 1 — The effect of different light qualities on the stomatal morphology of ‘Red Lion’ leaves at 28 d under 200 × and 2000 × magnification. [file Image_1.jpeg]

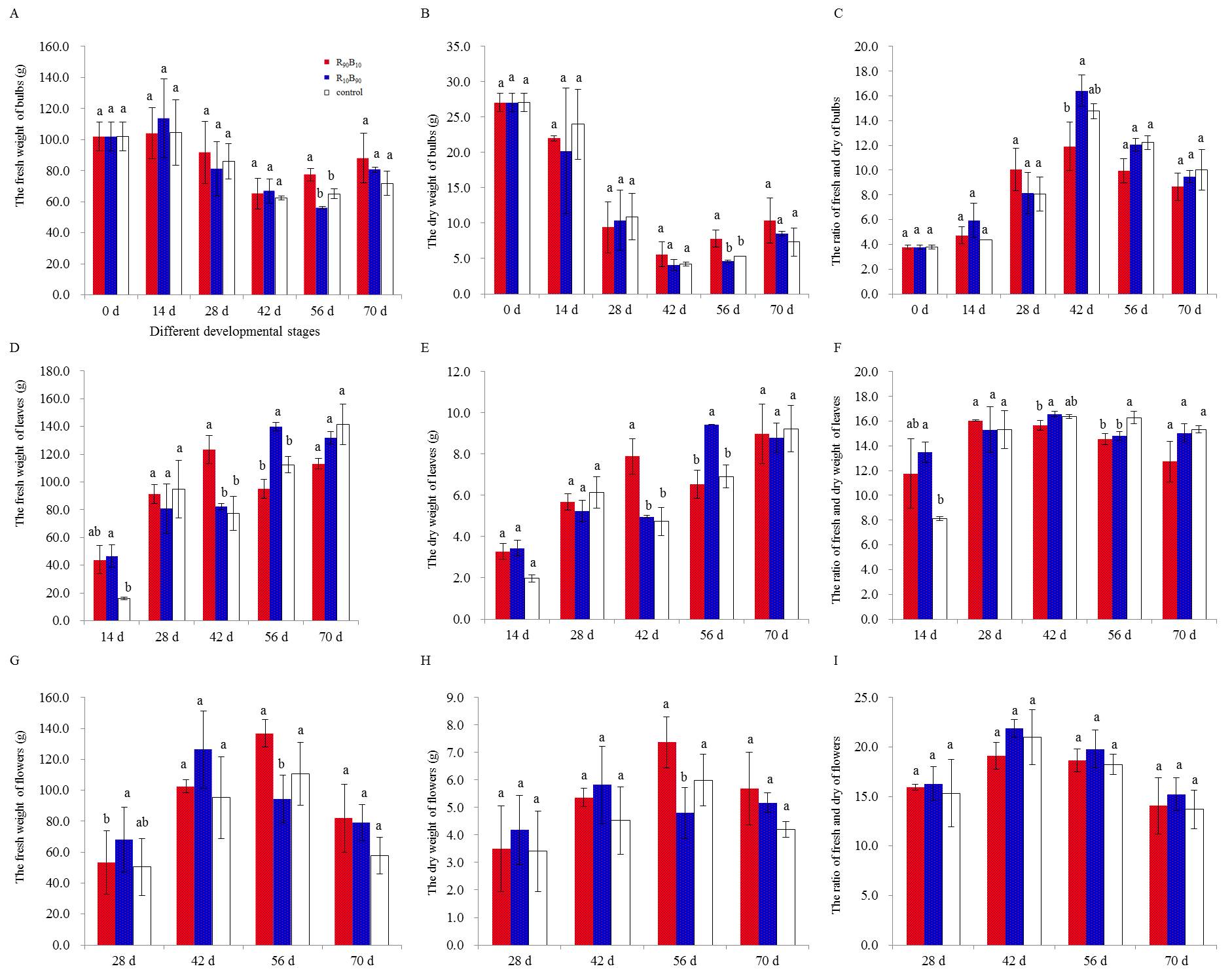

Supplement: Supplementary Figure 2 — The changes in fresh and dry mass and the ratio of the fresh and dry mass of bulb (A), leaf (B), and flower (C) under different blue and red light intensities. Different letters indicate significant differences at P< 0.05. [file Image_2.jpeg]

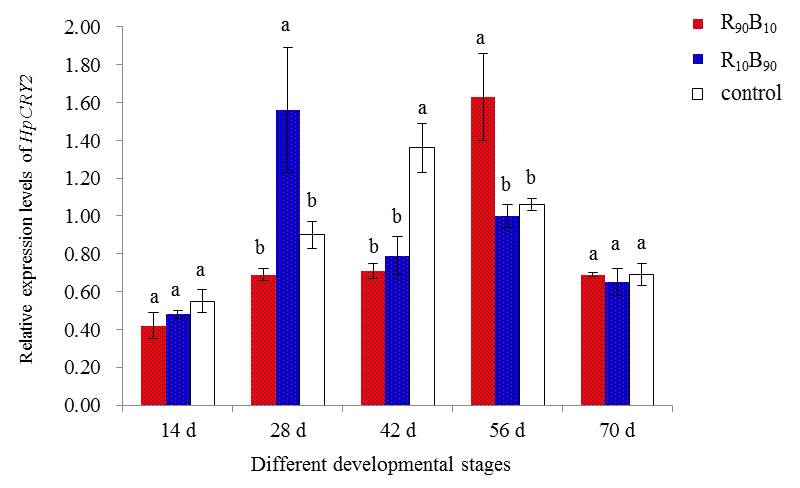

Supplement: Supplementary Figure 3 — The effect of different light qualities on the expression of the HpCRY2 gene in leaves. Different letters indicate a significant difference at P< 0.05. [file Image_3.jpeg]
