# Supplementary material for: The red/blue light ratios from light-emitting diodes affect growth and flower quality of Hippeastrum hybridum ‘Red Lion’
Source: Front Plant Sci. 2022 Dec 1;13:1048770. doi: 10.3389/fpls.2022.1048770 (PMC9751929; doi:10.3389/fpls.2022.1048770)
Supplement: Supplementary file 4 [file Table_1.docx]

**Table S1**

List of primers used for gene expression analysis for nine genes related to photosynthesis as well as flowering time and two reference genes

| Gene name | Primer name | The sequences of primer (5’-3’) | Length of the product (bp) |
| --- | --- | --- | --- |
| *HpEF-1α* | *HpEF-1αF* | GAGGCTGCTGAGATGAACA  CTGACCGTCCTTTGAGATAC | 264 |
|  | *HpEF-1αR* |  |  |
| *HpGAPDH2* | *HpGAPDH2F* | GCATTCAATCACTGCCACTCA  CACCTTGCCCACAGCCTTAG | 127 |
|  | *HpGAPDH2R* |  |  |
| *HpHEMA1* | *HpHEMA1F* | CGTGTCAGCGTTGGAGCA  GCTTTTCACGCATCTCTACAGG | 119 |
|  | *HpHEMA1R* |  |  |
| *HpHEML* | *HpHEMLF* | GGTTATTTCAGCCGTCCCT  TTGAGGACCTTCTGGCGA | 117 |
|  | *HpHEMLR* |  |  |
| *HpCHLH* | *HpCHLHF* | ACAGAGGCTTCTCGTGGA  AAACTTCGTCTTGGATAGGT | 150 |
|  | *HpCHLHR* |  |  |
| *HpCHLD* | *HpCHLDF* | TCTGGTAAGCGGGGAACT  CCGAGCGGAATCTGAACA | 212 |
|  | *HpCHLDR* |  |  |
| *HpCHLI* | *HpCHLIF* | TCTGTGACGAGCAAACCG  GAAGGGGAGGGATAAAGTG | 117 |
|  | *HpCHLIR* |  |  |
| *HpPOR* | *HpPORF* | CTTGCTTCTTCCCCTCAGTTC  GTGACAGTGGCGGTGGCT | 181 |
|  | *HpPORR* |  |  |
| *HpCAO* | *HpCAOF* | TTGCCAAGGGATGGAGTG | 170 |
|  | *HpCAOR* | CCTGGTATTTTGCCCCTC |  |
| *HpCRY2* | *HpCRY2F* | AAGGTTCTAAAGGGTTGGA | 121 |
|  | *HpCRY2R* | GACGAGGACTCAGCGGTA |  |
| *HpCOL* | *HpCOLF* | ATCTTGGAAGGTCTCGGGA | 161 |
|  | *HpCOLR* | AGGGCAGCCTGTGTCATA |  |
