# Supplementary material for: The red/blue light ratios from light-emitting diodes affect growth and flower quality of Hippeastrum hybridum ‘Red Lion’
Source: Front Plant Sci. 2022 Dec 1;13:1048770. doi: 10.3389/fpls.2022.1048770 (PMC9751929; doi:10.3389/fpls.2022.1048770)
Supplement: Supplementary file 5 [file Table_2.docx]

**Table S2**

The effect of different light qualities on the morphological leaf parameters of ‘Red Lion’

| Days | Treatments | No. of leaf | Length of leaf (cm) | Width of leaf (cm) | Length/Width |
| --- | --- | --- | --- | --- | --- |
| 14 d | R_90_B_10_ | 5.33 ± 0.67a | 19.71 ± 2.24a | 3.33 ± 0.21a | 5.82 ± 0.32a |
|  | R_10_B_90_ | 4.67 ± 0.33a | 17.95 ± 1.86a | 3.27 ± 0.21a | 5.37 ± 0.24a |
|  | Control | 4.67 ± 0.33a | 16.77 ± 2.48a | 3.08 ± 0.25a | 5.30 ± 0.40a |
| 28 d | R_90_B_10_ | 5.00 ± 0.58a | 43.04 ± 3.06a | 4.30 ± 0.23a | 9.91 ± 0.25a |
|  | R_10_B_90_ | 5.33 ± 0.33a | 37.77 ± 3.51a | 4.17 ± 0.33a | 8.97 ± 0.32a |
|  | Control | 5.67 ± 0.88a | 41.32 ± 1.94a | 4.26 ± 0.13a | 9.64 ± 0.25a |
| 42 d | R_90_B_10_ | 5.67 ± 0.33a | 55.72 ± 1.44ab | 4.58 ± 0.12a | 12.19 ± 0.46a |
|  | R_10_B_90_ | 5.00 ± 0.58a | 49.72 ± 2.20c | 4.78 ± 0.29a | 10.46 ± 0.27b |
|  | Control | 5.00 ± 0.58a | 52.81 ± 1.26ab | 4.45 ± 0.07a | 11.90 ± 0.43a |
| 56 d | R_90_B_10_ | 5.33 ± 0.67a | 58.49 ± 2.11ab | 4.57 ± 0.13a | 12.84 ± 0.65a |
|  | R_10_B_90_ | 6.33 ± 0.33a | 57.25 ± 2.04b | 4.98 ± 0.10b | 11.51 ± 0.48bc |
|  | Control | 4.67 ± 0.88a | 59.91 ± 1.18ab | 4.58 ± 0.18a | 13.18 ± 0.56a |
| 70 d | R_90_B_10_ | 5.67 ± 0.33a | 62.23 ± 2.64a | 5.00 ± 0.12a | 12.48 ± 0.43a |
|  | R_10_B_90_ | 5.67 ± 0.33a | 59.37 ± 2.28ab | 5.06 ± 0.12a | 11.79 ± 0.65a |
|  | Control | 5.67 ± 0.33a | 59.89 ± 2.15ab | 4.89 ± 0.06a | 12.27 ± 0.51a |

a, b, and c indicate significant differences at the P < 0.05 level.
