# Supplementary material for: The red/blue light ratios from light-emitting diodes affect growth and flower quality of Hippeastrum hybridum ‘Red Lion’
Source: Front Plant Sci. 2022 Dec 1;13:1048770. doi: 10.3389/fpls.2022.1048770 (PMC9751929; doi:10.3389/fpls.2022.1048770)
Supplement: Supplementary file 6 [file Table_3.docx]

**Table S3**

The effect of different light qualities on the morphological parameters of root and bulb development

| Days | Treatments | No. of root | No. of fibrous root | The longest length of root (cm) | Average length of root (cm) | Diameter difference of bulbs (cm)* |
| --- | --- | --- | --- | --- | --- | --- |
| 14 d | R_90_B_10_ | 20.33 ± 2.87aA | 7.33 ± 4.99aA | 18.83 ± 3.07aB | 10.07 ± 1.53aC | 1.70 ± 0.16aA |
|  | R_10_B_90_ | 26.67 ± 3.30aAB | 9.00 ± 5.35aA | 15.57 ± 0.68aC | 10.00 ± 0.78aA | 1.67 ± 0.40aA |
|  | Control | 21.67 ± 5.73aA | 5.33 ± 4.11aA | 17.50 ± 2.21aA | 9.90 ± 0.33aA | 1.43 ± 0.54aA |
| 28 d | R_90_B_10_ | 27.00 ± 2.83aA | 15.00 ± 2.83aA | 19.80 ± 1.06aB | 12.07 ± 0.29aBC | 1.03 ± 0.66aAB |
|  | R_10_B_90_ | 37.00 ± 8.98aA | 22.00 ± 8.83aA | 20.23 ± 1.89aBC | 12.80 ± 0.51aA | 0.50 ± 0.51aAB |
|  | Control | 24.00 ± 4.24aA | 14.00 ± 3.56aA | 20.53 ± 3.98aA | 12.50 ± 3.13aA | 0.90 ± 0.37aAB |
| 42 d | R_90_B_10_ | 23.00 ± 2.45aA | 20.67 ± 0.94aA | 28.20 ± 2.57aA | 12.17 ± 0.34aBC | -0.23 ± 0.25aBC |
|  | R_10_B_90_ | 21.33 ± 1.25aB | 18.33 ± 1.25aA | 27.73 ± 2.57aA | 12.60 ± 2.10aA | -0.13 ± 0.40aAB |
|  | Control | 24.00 ± 4.32aA | 18.67 ± 2.62aA | 22.37 ± 2.83aA | 14.77 ± 1.10aA | -1.57 ± 0.69aC |
| 56 d | R_90_B_10_ | 22.00 ± 2.83aA | 16.00 ± 2.45aA | 27.60 ± 2.02aA | 15.20 ± 0.90aA | -0.93 ± 0.40aC |
|  | R_10_B_90_ | 15.33 ± 4.11aB | 11.00 ± 6.16aA | 17.47 ± 2.08bBC | 12.03 ± 1.75aA | -0.57 ± 0.78aB |
|  | Control | 22.67 ± 3.30aA | 17.00 ± 4.97aA | 23.60 ± 3.16aA | 12.63 ± 0.29aA | -0.27 ± 0.77aABC |
| 70 d | R_90_B_10_ | 18.00 ± 5.35aA | 15.00 ± 7.12aA | 27.80 ± 3.61aA | 13.50 ± 0.41aAB | -0.80 ± 0.85aC |
|  | R_10_B_90_ | 15.67 ± 1.89aB | 11.67 ± 1.89aA | 26.77 ± 6.09aAB | 13.93 ± 1.10aA | 0.27 ± 1.03aAB |
|  | Control | 17.67 ± 5.25aA | 10.00 ± 6.38aA | 25.30 ± 0.65aA | 12.40 ± 0.86aA | -1.10 ± 1.02aBC |

The star represents the diameter difference of bulbs at 70 d and 0 d. Different lowercase and capital letters indicated a significant difference among different treatments (Duncan’s test at P < 0.05 after analysis of variance; data are shown as mean ± SD, n = 3).
