# Supplementary material for: The red/blue light ratios from light-emitting diodes affect growth and flower quality of Hippeastrum hybridum ‘Red Lion’
Source: Front Plant Sci. 2022 Dec 1;13:1048770. doi: 10.3389/fpls.2022.1048770 (PMC9751929; doi:10.3389/fpls.2022.1048770)
Supplement: Supplementary file 7 [file Table_4.docx]

**Table S4**

The effect of light qualities on flower color

| Treatments | *L^*^* | *a^*^* | *b^*^* | *C^*^* | *h^c^* |
| --- | --- | --- | --- | --- | --- |
| R_90_B_10_ | 36.81 ± 0.10a | 46.22 ± 3.07a | 30.68 ± 4.12a | 55.50 ± 4.84a | 33.42 ± 1.77b |
| R_10_B_90_ | 36.95 ± 1.66a | 47.76 ± 1.59a | 31.06 ± 1.72a | 56.97 ± 2.23a | 33.01 ± 0.69b |
| Control | 36.69 ± 1.61a | 48.32 ± 1.92a | 33.42 ± 1.97a | 58.76 ± 2.64a | 34.65 ± 0.74a |
