# Supplementary material for: The red/blue light ratios from light-emitting diodes affect growth and flower quality of Hippeastrum hybridum ‘Red Lion’
Source: Front Plant Sci. 2022 Dec 1;13:1048770. doi: 10.3389/fpls.2022.1048770 (PMC9751929; doi:10.3389/fpls.2022.1048770)
Supplement: Supplementary file 8 [file Table_5.docx]

**Table S5**

The effect of different qualities on the chlorophyll fluorescence parameters of ‘Red Lion’ leaves

| Developental days | Treatments | F_v_/F_m_ | Φ_PSⅡ_ | qN | qP | ETR | α |
| --- | --- | --- | --- | --- | --- | --- | --- |
| 14 d | R_90_B_10_ | 0.802 ± 0.006a | 0.063 ± 0.009ab | 0.675 ± 0.012b | 0.101 ± 0.015b | 48.133 ± 7.389b | 0.462 ± 0.036a |
|  | R_10_B_90_ | 0.807 ± 0.006a | 0.095 ± 0.024a | 0.785 ± 0.025a | 0.170 ± 0.045a | 68.833 ± 12.621a | 0.495 ± 0.027a |
|  | Control | 0.809 ± 0.008a | 0.055 ± 0.008b | 0.641 ± 0.014b | 0.084 ± 0.012b | 42.000 ± 6.18b | 0.392 ± 0.049b |
| 28 d | R_90_B_10_ | 0.817 ± 0.004a | 0.108 ± 0.022ab | 0.700 ± 0.047ab | 0.171 ± 0.037ab | 82.167 ± 16.955a | 0.495 ± 0.044a |
|  | R_10_B_90_ | 0.814 ± 0.006a | 0.120 ± 0.027a | 0.743 ± 0.01a | 0.199 ± 0.044a | 91.667 ± 20.136a | 0.492 ± 0.003a |
|  | Control | 0.812 ± 0.005a | 0.079 ± 0.015b | 0.671 ± 0.014b | 0.123 ± 0.025b | 59.800 ± 11.401b | 0.487 ± 0.023a |
| 42 d | R_90_B_10_ | 0.823 ± 0.003a | 0.128 ± 0.031ab | 0.699 ± 0.017a | 0.215 ± 0.034a | 89.167 ± 11.714b | 0.452 ± 0.031b |
|  | R_10_B_90_ | 0.827 ± 0.005a | 0.144 ± 0.001a | 0.722 ± 0.018a | 0.234 ± 0.002a | 109.800 ± 0.529a | 0.459 ± 0.053ab |
|  | Control | 0.819 ± 0.005a | 0.087 ± 0.006b | 0.682 ± 0.051a | 0.125 ± 0.012b | 66.000 ± 4.451c | 0.517 ± 0.029a |
| 56 d | R_90_B_10_ | 0.825 ± 0.006a | 0.115 ± 0.018b | 0.765 ± 0.025a | 0.192 ± 0.033b | 87.533 ± 13.937b | 0.431 ± 0.052a |
|  | R_10_B_90_ | 0.824 ± 0.002a | 0.161 ± 0.009a | 0.760 ± 0.029a | 0.270 ± 0.015a | 122.467 ± 6.643a | 0.472 ± 0.026a |
|  | Control | 0.817 ± 0.021a | 0.09 ± 0.01b | 0.681 ± 0.063b | 0.139 ± 0.02b | 68.533 ± 7.445c | 0.485 ± 0.039a |
| 70 d | R_90_B_10_ | 0.810 ± 0.008ab | 0.161 ± 0.011a | 0.736 ± 0.014ab | 0.268 ± 0.02a | 122.733 ± 8.444a | 0.408 ± 0.038b |
|  | R_10_B_90_ | 0.823 ± 0.006a | 0.131 ± 0.023ab | 0.787 ± 0.018a | 0.232 ± 0.04a | 101.767 ± 14.19b | 0.384 ± 0.039b |
|  | Control | 0.810 ± 0.002b | 0.099 ± 0.04b | 0.707 ± 0.034b | 0.156 ± 0.062b | 64.033 ± 11.102c | 0.488 ± 0.01a |

Different small letters indicate significant differences at the P < 0.05 level.
